# Apple Leaf Disease Detection Based on Improved YOLOv11 with DSSA Mechanism

**DOI:** 10.3390/plants15121928

**Published:** 2026-06-22

**Authors:** Yuanyuan Zhang, Jiya Tian, Duanyang Zhang

**Affiliations:** College of Information Engineering, Xinjiang Institute of Technology, Aksu 843000, China; 2022167@xjit.edu.cn (Y.Z.); 2020032@xjit.edu.cn (J.T.)

**Keywords:** apple leaf disease, accurate disease identification, YOLOv11, Dual Sparse Selection Attention (DSSA), visualization system

## Abstract

Visual inspection of apple leaf diseases is inefficient and subjective, limiting large-scale orchard applications. To realize rapid and accurate disease identification, this paper proposes an improved YOLOv11 model integrated with a Dual Sparse Selection Attention (DSSA) module. By embedding the DSSA module into the key layers of the YOLOv11 backbone network, the model enhances fine-grained feature extraction for small and complex lesions while suppressing background interference. A tailored training strategy with an optimized learning rate and optimizer is designed to ensure stable convergence. Experiments are conducted on a dataset consisting of 7594 images covering four categories: black rot, rust, scab, and healthy leaves. The proposed model achieves precision of 0.973, recall of 0.978, mAP50 of 0.991, and 0.949 mAP50–95, outperforming YOLOv8, YOLOv9, YOLOv10, and the vanilla YOLOv11. Furthermore, a Qt-based visualization system is developed for practical orchard deployment. This method provides a reliable solution for intelligent apple leaf disease detection and smart orchard management.

## 1. Introduction

Apples planted in Aksu, Xinjiang, are an important economic crop and a pillar industry that supports the income of local farmers and rural revitalization. Leaves organs are very important for photosynthesis and nutrition transportation; their health condition directly determines the output and quality of apple trees. The main leaf diseases, which include black rot, rust and scab, spread rapidly [1,2]. Delayed detection and control of these diseases can reduce crop yields by 20–50% and even cause complete crop loss when infections are serious [3,4].

The traditional check by apple leaf disease inspectors mainly depends on visual and expert experience, which has three crucial shortcomings:

1. Low working efficiency: One single person can check only 5–8 mu (≈3333.35 m^2^–5333.36 m^2^) of fruit garden per day; therefore it is hard to fit into large-scale planting modes.
2. High subjectivity: Inspectors have clear differences in judging early sickness signs, with misdiagnosis rates reaching as high as 15–25%.
3. High technical barriers: Basic agriculture stations and small planters lack technical workers, leading to delayed diagnosis and treatment.

With the rapid development of computer vision and deep learning, intelligent detection methods based on object detection algorithms have already become a mainstream solution for the above problems [5,6,7,8]. These methods have the characteristics of fast detection speed, high accuracy and good repeatability; therefore they can realize early, accurate batch recognition of apple leaf diseases.

The YOLO series is extensively utilized in agricultural object detection on account of its fine real-time property and efficient inference process. Nevertheless, in the detection of apple leaf diseases, there exist challenges including small disease spot regions, complicated texture features, and background disturbance (e.g., leaf overlapping and non-uniform illumination) which are still difficult to deal with. The vanilla YOLOv11 has difficulty with fine-grained feature extraction and background suppression, leading to comparatively low precision on small and fuzzy lesions, and it cannot completely capture key disease features [9,10].

Attention mechanisms can assist the model in focusing on key regions and filtering out redundant information, which effectively promotes detection precision. The Dual Sparse Selection Attention Mechanism (DSSA) utilizes a double sparse strategy to find the core feature parts, and thus it displays stronger distinguishing capability in complicated environments [11]. Therefore, this research integrates the DSSA module into the basic framework of YOLOv11 to construct an improved model for apple leaf disease identification, with the aim of overcoming the performance restrictions of current methods.

In recent years, agricultural disease detection has gradually evolved from traditional machine vision to deep learning methods. Early methods that depend on hand-made features such as LBP and HOG [12,13], which are combined with classifiers that include SVM and random forest [14,15], thus display restricted capacity to adapt to complex backgrounds and various disease symptoms. Deep learning has greatly improved detection accuracy through end-to-end characteristic study. For example, a model based on CNNs achieved 89.7% accuracy in the classification of apple leaf diseases. Faster R-CNN achieved an mAP50 of 0.90 in apple scab detection, but its inference speed of only 22 FPS was not able to satisfy real-time requirements. Structures based on Transformers can promote the representation of global features, but they bring excessive computation, making them unsuitable for low-cost field devices [16,17,18].

Since YOLO was proposed in 2015, the YOLO series has been promoted through 11 editions, with continuous promotions in both detection accuracy and efficiency. The C3k2 module is used by YOLOv11 for the replacement of C2f, and it also integrates the C2PSA spatial attention mechanism. It can raise mAP, while cutting down parameters by 22 percent, and supports detection, segmentation, and pose estimation. Improved YOLO models have already been widely utilized in agricultural disease monitoring: an enhanced YOLOv8 detected rice blast disease with 0.92 mAP50 under 35 FPS; and a YOLOv9 model embedded with ECA attention realized 0.89 mAP50-95 on corn borer detection [19,20].They struggle with picking out fine and complicated damage features, and do not have practical project arrangement, making it difficult to meet real orchard managing demands. Three main challenges still remain:

1. Insufficient extraction of fine lesion features, which causes low sensitivity for small and early illness.
2. Lack of practical engineering applications, with few friendly vision tools for common planting farmers and agriculture workers.
3. Poor anti-interference capability in complex field environments, with effects clearly dropping under non-uniform illumination and leaf overlap.

Hence, there is an urgent necessity for developing a high-precision, real-time, convenient apple leaf disease identification system to support the development of intelligent agriculture.

The Dual Sparse Selection Attention (DSSA) utilizes combined channel and space sparse filtration to eliminate redundant information and emphasize important disease characteristics. It has achieved good performance in image classification and object detection, but has not seen systematic application in apple leaf disease identification. Taking into account the unique feature distribution that apple leaf diseases have, this research inserts the DSSA module into the YOLOv11 backbone network and optimizes the training strategy, promoting detection accuracy and robustness at the same time. Consistent with the comprehensive overview from Ubbens et al. [21], lightweight YOLO integrated with attention has become a mainstream technical route in modern plant phenotyping research.

The main contribution of this study can be summarized in three parts:

1. We construct an enhanced YOLOv11 + DSSA model through inserting the DSSA module into the key layers of the YOLOv11 backbone network, with the purpose of promoting its fine-grained feature extraction ability.
2. We design a targeted training plan that is fit for the DSSA module, which includes learning rate modification and optimizer choice, to guarantee stable convergence in the training process.
3. We carry out enough comparison experiments on the disease dataset of apple leaves to verify the performance superiority of the improved model and thus analyze its actual detection effect.
4. We develop a visual Qt system for real-world orchard application.

## 2. Materials and Methods

### 2.1. Foundation of YOLOv11 Model

YOLOv11 uses a module-based design that has three core components: backbone, neck, and head. It adopts an anchor-free architecture, can give support to many vision tasks, and carries out the use of parameters in an efficient way [22].

The backbone is in charge of extracting features from input images, and it produces multi-scale feature maps through convolution and residual connections. To speak concretely, the backbone of YOLOv11n contains convolution layers, C3k2 residual blocks, the SPPF module, and the C2PSA attention mechanism. SPPF carries out the fusion of multi-scale spatial information and expands the receptive field; hence C2PSA enhances feature expression by focusing on critical regions.

The neck component carries out the fusion of multi-scale features that come from the backbone module. By means of upsampling and concatenation operations, deep semantic features and shallow high-resolution details are combined by it to enrich feature diversity, and thus it provides more effective feature representation for detection.

The head utilizes an anchor-free detector to directly forecast bounding boxes, confidence scores, and category probability distributions from the integrated features. This method has the support to realize detection on multiple different scales; therefore it is suitable for locating disease spots with different sizes that exist on apple leaves.

### 2.2. Principle of DSSA Module

DSSA is a kind of lightweight attention mechanism that is designed for vision-related tasks. It picks out task-connected core characteristics and reduces computational cost by means of a double sparse method on both region and pixel levels, which effectively suppresses background noise and redundant information.

The DSSA module contains three important components: region-level sparse token choosing, pixel-level sparse token choosing, and output feature computation.

In the selection of region level, the input feature map with a size of H × W × C is cut into S × S grid areas. Matrix multiplication is utilized to calculate the similarity between the region query vector q^r^ and each region key vector kir, generating the region attention score matrix A^r^. The regions that have top-*k*_1_ maximum scores are kept, while non-related background regions are thrown away, and hence the calculation burden is decreased.

Pixel-level selection: For the kept areas, the pixel-grade query Q is utilized to compute the similarity with the pixel keys K^g^ to get the pixel attention score matrix A^p^. The top-*k*_2_ pixels that possess the highest scores are retained, further suppressing the background noise inside the regions and enhancing the lesion features.

Output computation: The kept pixel value features V_gg_ are multiplied by the normalized attention weights (after softmax) to produce the final strengthened feature O, which is sent into the following neck network for object detection.

**Figure 1:**
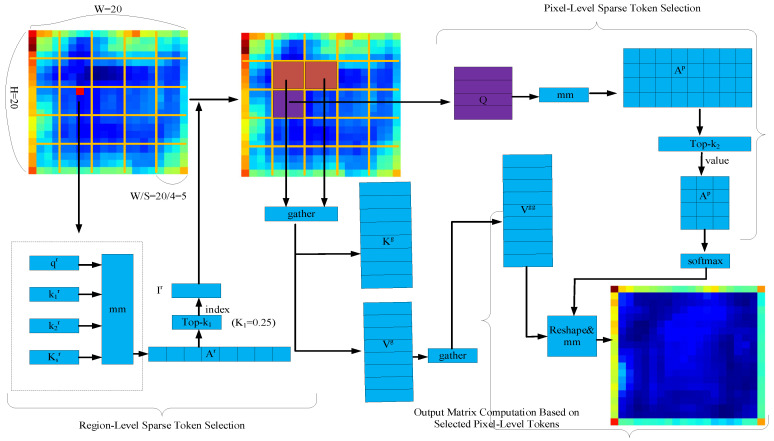
Workflow of the proposed Dual Sparse Selection Attention (DSSA) module. This module takes 20 × 20 feature maps output by the SPPF layer and adopts dual sparse screening with *k*_1_ = 0.25 and *k*_2_ = 0.5 for apple leaf disease detection.

The detailed working flow of the DSSA module that we put forward for apple leaf disease inspection is shown in Figure 1. The DSSA module uses a 20 × 20 feature map (output from the SPPF module) as its input.

1. Region-level sparse token choice: The feature map is cut into 5 × 5 areas when S = 4, and the top-*k*1 = 0.25 high-importance areas that have lesions are kept through similarity computation. The gather operation takes out the feature information of these regions to produce Kg and Vg.
2. Pixel-level sparse token choice: For the kept regions, we compute pixel-level attention scores and retain the top 50% of (*k*_2_ = 0.5) pixels with the highest significance. The attention weight values are then normalized through utilization of the softmax function.
3. Result calculation: The ultimate 20 × 20 strengthened feature map is acquired through weighting the chosen pixel features with the normalized attention scores. Using this method, the lesion features are obviously enhanced, and hence the background interference is effectively restrained.

### 2.3. Overall Structure of the Improved Model

**Figure 2:**
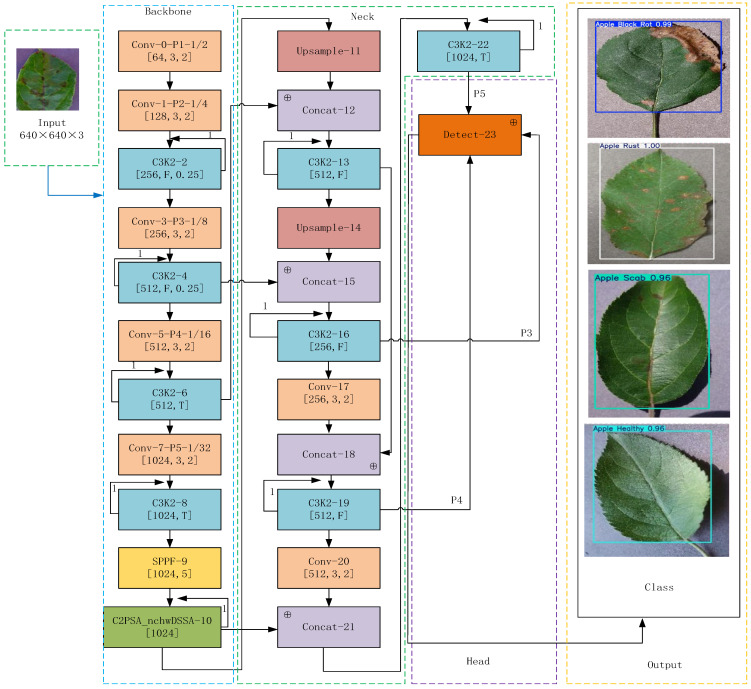
Overall architecture of the improved YOLOv11 + DSSA model. The DSSA module is embedded after the SPPF layer in the YOLOv11 backbone to enhance lesion feature extraction.

The improved YOLOv11 + DSSA model retains the regular three-section composition: backbone, neck, and head. Its main promoted point is that it adds the C2PSA_nchwDSSA-10 module at the last position of the backbone. This module adopts a double sparse screening method (which includes region level and pixel level) to better extract characteristics that are related to leaf diseases. The whole structure of the network is displayed in Figure 2.

Before the step of feature extraction, input images are resized to 640 × 640 × 3 and then sent to the backbone. For the promotion of generalization ability, data augmentation, which includes random flipping and cropping, is utilized in the pre-processing step.

The backbone makes a multi-scale high-level feature map by step-by-step downsampling and feature study:

1. Conv-0 downsamples the input with stride 2 to produce the P1 feature map at 1/2 size.
2. Conv-1 further downsamples P1 to get the P2 feature map at 1/4 size.
3. C3k2-2 uses two small convolutions in place of one big convolution to enrich the semantic information of P2, and Conv-3 then outputs the P3 feature map at 1/8 size.
4. C3k2-4 draws fine-grained details from the P3 feature map, laying a basis for later high-level feature learning; after that, Conv-5 reduces the size of P3 to produce the P4 feature map under 1/16 scale.
5. C3k2-6 brings in residual connections to enhance semantic connection and stability of the P4 feature map.
6. Conv-7 carries out size reduction to produce the P5 feature map under 1/32 scale, which holds the richest high-level semantic information.
7. SPPF-9 carries out multi-scale spatial pyramid pooling on the P5 feature map, fuses spatial messages from different scales, restrains partial noise, and outputs a steady 1024-dimension feature map.

As the core promoted component, the C2PSA_nchwDSSA-10 module takes the 1024-dimensional features from SPPF-9 and carries out double sparse filtering (region and pixel levels), with fixed *k*_1_ = 0.25, *k*_2_ = 0.5 as defined, and the optimized features are directly transmitted into the subsequent neck layer without modifying original network structure.

This step makes features related to disease stronger, and at the same time it suppresses background information which has no use.

The neck component utilizes a feature pyramid network (FPN) to carry out multi-scale feature fusion work. It is combined with upsampling, channel connection, and C3k2 blocks for merging deep semantic features and shallow high-resolution details, making fused features have more distinct characteristics and more information. Finally, the neck module gives out three strengthened feature maps: F3 (1/8 proportion), F4 (1/16 proportion), and F5 (1/32 proportion), which are utilized by people to inspect small, medium, and large damage areas one by one.

The detection head carries out multi-branch detection on the basis of the three feature maps that come from the neck. Every measuring scale corresponds to one independent separate branch. Features get purified by the CBS module, and the Conv2d layer gives out forecast outcomes which include bounding box coordinates, confidence scores, and class probabilities. For the target screening work, the IoU threshold value is set to 0.5.

The loss function that the YOLOv11 + DSSA model uses includes three component parts: box loss, classification loss, and distribution focal loss. When combined, they carry out optimization of the model’s positioning accuracy and classification performance, thereby ensuring that detection results are stable and reliable.

## 3. Results

### 3.1. Experimental Environment

All experiments were conducted on a standard computer that possesses an Intel Core i5 CPU, an NVIDIA GTX 1050 4G GPU, and 16 GB of RAM. The system runs on Windows 10. Our research employed Python edition 3.8, PyTorch edition 1.18, and the Ultralytics framework (edition 8.0 or higher), together with NumPy2.1.3 and Matplotlib3.10.0, to carry out data handling and image displaying. All inference speed tests were carried out on an identical hardware platform. The model that we put forward obtained 32 FPS on this platform, satisfying the real-time requirements of orchard detection. The total training time for all models was about 12 h using this hardware. The limited 4G VRAM of GTX1050 results in a small batch size of 2; the DSSA lightweight sparse design avoids excessive computation costs, so 32 FPS real-time inference can be realized on a single 640 × 640 input.

### 3.2. Dataset Introduction

In the present research, we utilize a specialized dataset constructed for apple leaf disease identification. It includes four categories: apple black rot, healthy leaves, apple rust, and apple scab. This dataset contains many different types of scenes with diverse lighting conditions, shooting angles and complex background circumstances. It also contains difficult test samples such as extremely small damaged areas (pixel proportion < 5%) and fuzzy organization forms. All images are resized to 640 × 640 and marked in the standard YOLO form. The data collection is divided into training, verification and testing, with an approximate proportion of 7:2:1. Distribution details are enumerated in Table 1. All images were collected from multiple independent orchards, and samples from the same fruit tree were randomly distributed across train/val/test sets to avoid data leakage. The various augmentations listed in Section 3.3 compensate for the dataset size limitation.

### 3.3. Training Strategy Optimization

All comparison models adopted identical input size, epoch, batch and augmentation rules; the poor results of the original YOLO series were caused by numerous tiny lesions and complex field backgrounds in our collected dataset.

In order to match the parameter characteristics of the DSSA module and guarantee an equitable comparison with the original YOLOv11, we have carried out optimization on the training settings, which are enumerated as follows:

Basic constitution settings: The model carries out training for 50 cycles, with a batch size of 2, and the input picture size is fixed at 640 × 640, which is consistent with the official YOLOv11 arrangement.

Learning rate: The initial learning rate is set to 5.0 × 10^−5^ in order to avoid the occurrence of gradient explosion. The learning rate decline coefficient is 0.01, and eight warm-up periods are utilized to allow early training stabilization. This small initial learning rate is selected after repeated preliminary tests to prevent parameter shock from the newly added DSSA module.

Optimizer: We utilize the AdamW optimizer (not SGD in YOLOv11), which has a momentum of 0.937 and a weight decay of 0.001, for the enhancement of training stability and the reduction of overfitting.

Data expansion: Mosaic (0.8), horizontal flip (0.5), HSV intensification, small rotation (5.0°), and position shift (0.1) are utilized to promote generalization ability. Random erasing is disabled, to avoid damaging tiny lesion features.

### 3.4. Evaluation Metrics

Five common metrics in object detection are used for quantitative assessment:

Precision (*P*)—The ratio of correctly detected positive samples to all predicted positive samples:

(1) $$P=\frac{TP}{TP+FP}$$

Recall (*R*)—The ability of the model to reduce missed detections:

(2) $$R=\frac{TP}{TP+FN}$$

*F*1-Score—The harmonic mean of precision and recall, indicating balanced overall performance:

(3) $$F1=2\times \frac{P\times R}{P+R}$$

mAP_50_ refers to the mean average precision at an IoU threshold of 0.5, serving as the core evaluation metric.

mAP_50–95_ represents the average AP calculated with IoU thresholds from 0.5 to 0.95 (step = 0.05), which therefore reflects the ability of precise object localization.

These measurement indexes comprehensively reflect the model’s precision, recall rate, localization accuracy, and overall detection performance level.

### 3.5. Analysis of Training Process

According to the training curves and experiment results, the YOLOv11 + DSSA model in the training process manifests the following behaviors.

**Figure 3:**
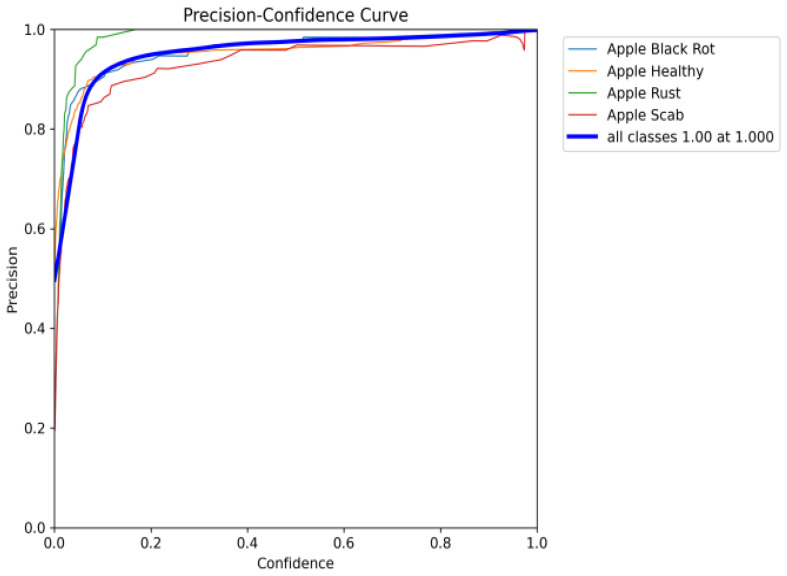
Precision curves of four apple leaf disease categories under different confidence thresholds.

As shown in Figure 3, the precision–confidence curve has proven that good overall and single-class performance is achieved. When the confidence threshold is larger than 0.2, the precision value of every category rapidly goes beyond 0.8 and remains at a high level along with the increase in confidence. When the threshold value surpasses 0.5, the precision stabilizes between 0.95 and 1.0. The curves of all classes converge to 1.0, indicating that the YOLOv11 + DSSA model can provide highly dependable detection with few wrong predictions.

**Figure 4:**
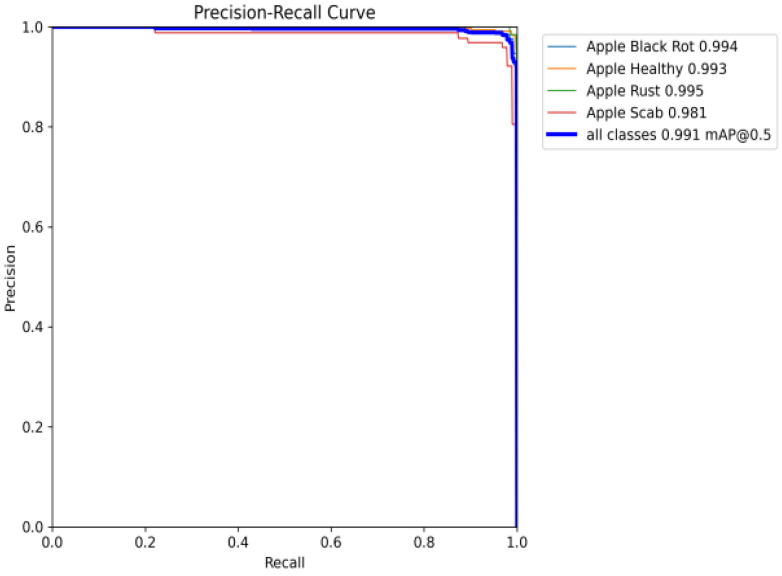
Precision–recall curves for each category as well as the overall mAP@0.5.

In Figure 4, we present the precision–recall curves with the AP for every class and the total mAP@0.5. All curves are positioned near the top-right corner, which means the false positive rate and false negative rate are both extremely low. The AP numerical values of apple black rot (0.994), healthy apple (0.993), and apple rust (0.995) are almost 1.0, which suggests that the detection performance is nearly perfect. Apple scab produces a slightly lower AP value of 0.981, but it still stays on a high level. The whole mAP@0.5 achieves 0.991, which shows that this model possesses strong whole detection ability. The dual sparse screenings in DSSA effectively highlight lesion features, promote discrimination between different disease types, and increase the recognition of small and blurred lesions, leading to fine precision and recall at the same time.

**Figure 5:**
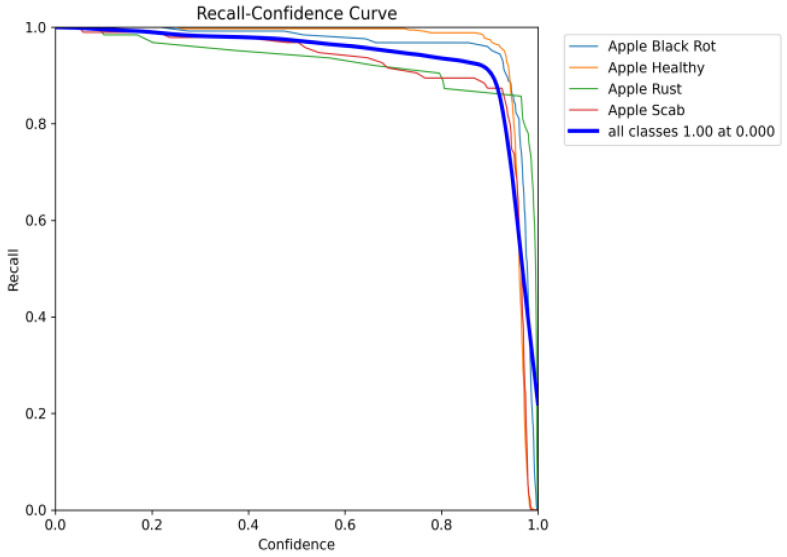
Recall curves of all tested categories across varying confidence levels.

The recall–confidence curve is displayed in Figure 5. When the confidence threshold is lower than 0.8, the recall value of all classes remains above 0.95, meaning that the missed detection rate is extremely low. When the threshold approaches 1.0, the recall value quickly drops, because high-confidence configurations filter out uncertain outcomes. The “all classes” curve attains 1.0 at a near-zero threshold, providing proof that real samples can be completely covered. The DSSA module enhances lesion features and supports accurate and complete detection, thus satisfying the actual demands of low false detection rates and low missed detection rates.

**Figure 6:**
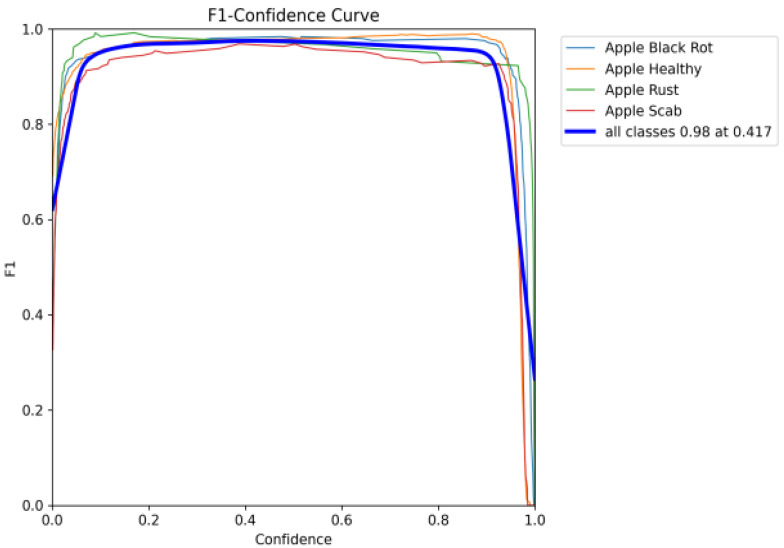
F1-score curves of all categories at different confidence thresholds.

The *F*1–confidence curve is displayed in Figure 6. The model that we built maintains a high *F*1-score across a wide range of confidence threshold values. In the interval from 0.1 to 0.8, the *F*1-score stays higher than 0.95, and the curves of different categories show very high consistency, demonstrating that precision and recall are in a balanced state, with no obvious bias. The curves of all classes reach a peak value of 0.98 when the confidence threshold is 0.417, remaining near 1.0 from 0.2 to 0.7; this therefore supports that it can have stable use in actual situations. The *F*1-score rapidly drops when the confidence threshold approaches 1.0.

**Figure 7:**
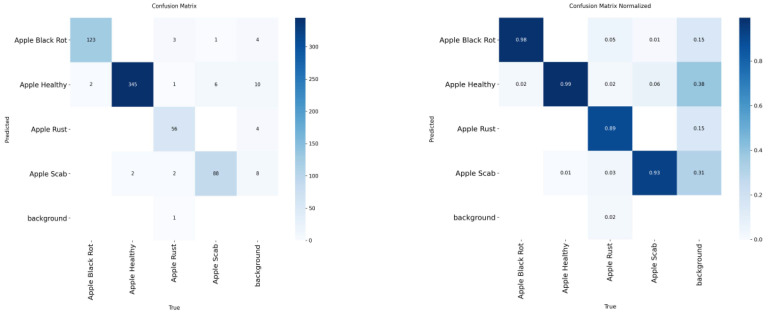
Original and normalized confusion matrix for four apple leaf disease categories.

Figure 7 displays the confusion matrix and its normalized form. Both sides reflect extremely low classification error rates among these four categories. Only a little confusion appears between healthy leaves and apple scab, which therefore fully meets the accuracy requirements of actual detection.

**Figure 8:**
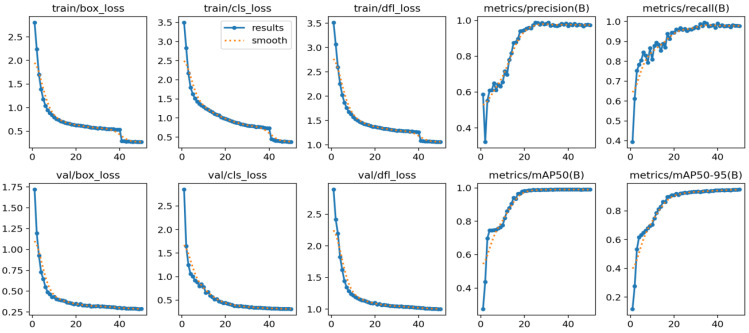
Changes in training loss, validation loss, precision, recall, mAP50 and mAP50–95 of the proposed model over 50 training epochs.

Figure 8 shows the training loss curve and the validation loss curve, together with the curves of key evaluation indicators. The training loss can reach steady state after approximately 20 training cycles, a phenomenon that reflects fast convergence speed. The validation loss drops in accordance with the training loss and remains at a comparable level, meaning that the model does not have an overfitting problem and possesses good generalization ability. The precision value increases from 0.4 to higher than 0.95 and gradually stabilizes near 1.0. The recall value also increases from 0.4 to above 0.95, maintaining a high numerical value. The value of mAP_50_ increases from 0.2 to above 0.95, and it stays near 1.0. mAP_50–95_ increases from 0.2 to higher than 0.8, which demonstrates that accurate position locating can be realized even under strict IoU threshold conditions.

The model we have constructed achieves effective learning over the course of 20 epochs and presents no overfitting issues, demonstrating stable high performance on all of the main metrics. These outcomes prove the usefulness of the YOLOv11 + DSSA structure which we put forward.

## 4. Discussion

### 4.1. Horizontal Comparison Experiment

**Figure 9:**
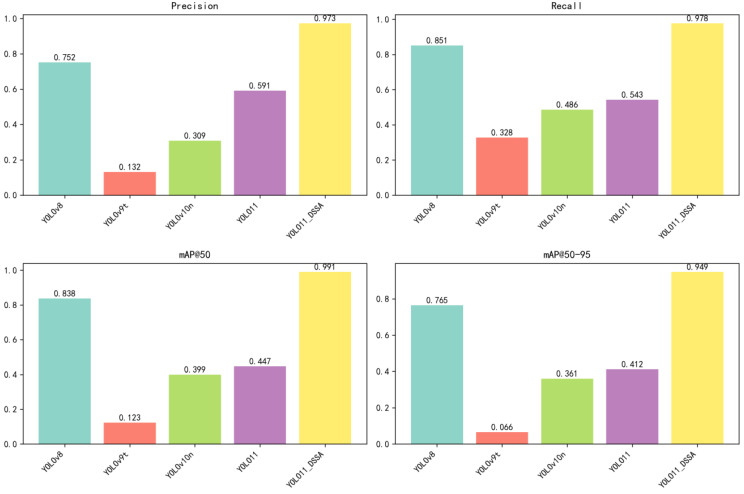
Comparison of precision, recall, mAP50 and mAP50–95 for YOLOv8, YOLOv9t, YOLOv10n, vanilla YOLOv11 and the proposed YOLOv11 + DSSA on the test set.

All comparative models were trained with the same settings for fairness. We have made a comparison of core indicators between the YOLOv11 + DSSA model and a number of mainstream models of the YOLO series on the test set, and the results are presented in Table 2 and Figure 9.

Figure 9 displays bar graphs that compare some YOLO series models for apple leaf disease detection, using four important norms: precision, recall, mAP50, and mAP50-95. This comparison was carried out quantitatively verify that our improved YOLOv11 + DSSA model has better performance than current mainstream YOLO versions. In this paper we prioritize lightweight YOLO variants matching portable field deployment; comparisons of RT-DETR and Faster R-CNN will be completed in future research.

Five types of models have been tested: the YOLOv8, the YOLOv9, the YOLOv10n, the original YOLOv11, and our proposed YOLOv11 + DSSA. According to the figure, YOLOv11 + DSSA obtains the best outcomes on all measurement indexes, and a detailed analysis is carried out below:

Precision degree: The YOLOv11 + DSSA model attains 0.973, which is significantly higher than all the other models. This proves that the upgraded model possesses higher prediction accuracy and hence generates fewer false positive results when it carries out the identification of apple leaf diseases.

Recall: The model achieves a recall value of 0.978, which clearly exceeds all the other methods. This shows that it possesses a strong capability for identifying true positive samples, and hence it can effectively reduce missed detections.

mAP50: The YOLOv11 plus DSSA method achieves a mAP50 of 0.991, but among the other models, the highest value, that of YOLOv8, is only 0.838. This great disparity proves that our model has significantly stronger ability in two aspects, target position finding and category division.

mAP50-95: When the model has an mAP50-95 value of 0.949, it maintains very good detection ability even when it works under strict IoU threshold values from 0.5 to 0.95.

To conclude, for the detection of apple leaf diseases, YOLOv11 + DSSA has better performance than YOLOv8, YOLOv9, YOLOv10n, and the original YOLOv11. Through the embedding of the DSSA module, the network’s capability for feature extraction and filtering obtains very good enhancement, resulting in a distinct performance improvement compared with the baseline YOLOv11. Hence, the proposed YOLOv11 + DSSA model meets the actual needs of high-precision, high-recall disease detection in large orchard surroundings. The DSSA module greatly enhances inspection accuracy.

### 4.2. Visual Real-Time Detection

**Figure 10:**
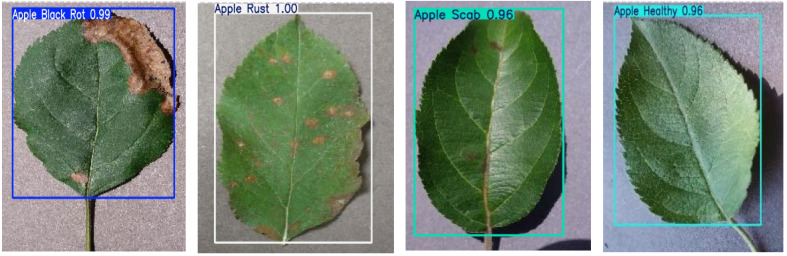
Visual detection results of typical apple leaf samples with various diseases.

Figure 10 displays the real-time visual inspection outcomes of the model on test samples. Based on this visualization, the model we put forward can accurately carry out location and classification on both healthy leaves and all kinds of disease types. The boundary boxes are well aligned with the real pathological change regions, having no evident position deviation or undetected situations. Furthermore, the model carries out work in a stable way under complex background conditions; it effectively separates target leaves from objects that cause interference and focuses on key lesion areas.

### 4.3. Apple Leaf Disease Detection System

The Apple Leaf Disease Inspection System utilizes a modular Qt-based graphical interface comprising three core function modules that are defined in UI files. These modules constitute one entire workflow that includes user login, multi-mode disease check, and data handling. (1) User Authentication: This step supports user sign up and login checking to guarantee the safety of system resources and past check records. (2) Multi-Modal Disease Check: This step offers four check modes to fit different agriculture usage situations.

**Figure 11:**
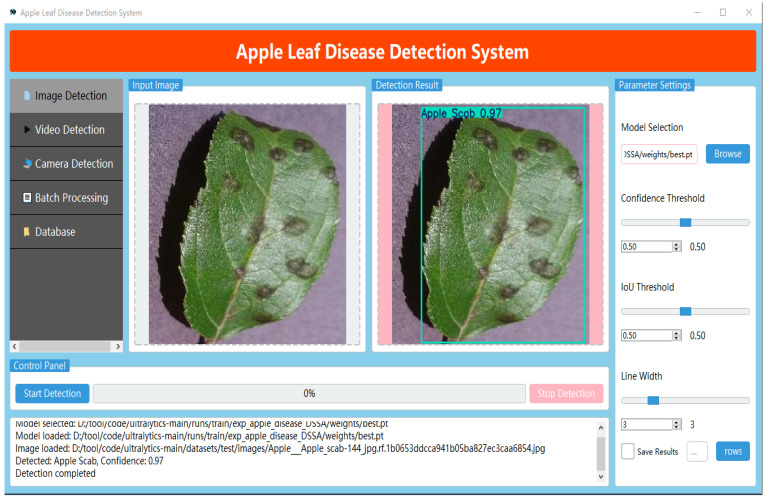
Interface demonstration of the Qt-based apple leaf disease detection system.

Figure 11 shows a complete running process of the system through the main interface.

Input and Handling: The left-side panel shows the original input picture of an apple tree leaf. The system as completes the correct loading of the pre-trained YOLO model and the test image, just as the log output at the bottom shows.

Result of Detection: The right-side panel displays the final output of detection. This model can correctly find the position of the lesion by using a bounding box, and therefore categorizes the lesion as apple scab with a confidence value of 0.97, which reflects that this model has relatively strong performance in classification and localization.

System Condition: The control panel shows that detection has already been finished. The parameters on the right side are set to default values (confidence = 0.50, IoU = 0.50), proving that the system can operate stably under the standard configurations.

This visual experiment confirms that the system, by means of a friendly and direct-viewing interface, realizes high-accuracy disease identification, thus providing users with clear and usable outcomes.

## 5. Conclusions

### 5.1. Research Conclusions

This study proposes an improved YOLOv11 model combined with the Dual Sparse Selection Attention (DSSA) mechanism for the detection of apple leaf diseases. By inserting the DSSA module into the backbone of YOLOv11, the model carries out dual sparse filtering for lesion features, which greatly enhances its capability for catching small and complex lesion areas. At the same time, the training strategy is carefully regulated, including the learning rate and the choice of optimizer, to guarantee stable convergence of the model and higher efficiency of training.

Experiments conducted on a specialized apple leaf disease dataset show that the YOLOv11 + DSSA model obtains 0.973 on the precision index, 0.978 on the recall index, 0.991 on mAP50, and 0.949 on mAP50–95. These obtained outcomes are distinctly superior to those of the initial YOLOv11 and other current mainstream YOLO detectors, for instance, YOLOv8, YOLOv9, and YOLOv10n. Additionally, the model retains balanced recognition accuracy across four kinds of apple leaves, with low false detection rates and strong capability of generalization. This work proposes a usable high-accuracy method for intelligent disease assessment and observation in fruit gardens, and therefore can also work as a reference for enhancing similar target detection models in agricultural applications.

### 5.2. Limitations and Future Work

Although the experiment’s outcomes are satisfying, the present research still has some restrictions that need to be solved in the future work.

Firstly, the data collection only includes four ordinary apple leaf kinds: apple black rot, healthy leaf, apple rust, and apple scab. The model’s performance on rare apple diseases has not been completely verified; hence it needs more diverse samples.

Second, the model’s firm anti-interference ability under extreme light conditions—including strong direct sunlight and weak light—and serious shelter situations (weed occlusion, serious leaf overlapping) requires further promotion to better fit complex farmland environments.

Third, because people bring in the DSSA module, the inference speed shows a small reduction, which makes it hard to completely satisfy the need for field real-time detection in large-scale fruit gardens.

To address these limitations and support real-world application, therefore, future studies will focus on the following aspects:

Expand the dataset and increase the number of disease categories, adding rare diseases and samples under complex field conditions such as different leaf growth stages and weather changes, to promote the model’s adaptation ability.

Use network pruning, quantization and other technologies to optimize the lightweight structure of the DSSA module, accelerating the inference process while maintaining a high detection accuracy.

Through the transfer learning method, we extend the YOLOv11 + DSSA model to disease detection for other fruit trees, including pears, peaches, and cherries, thus realizing cross-crop generalization ability and a wider application range.

## Figures and Tables

**Table 1:** Dataset scale distribution.

Category	Number of Training Images	Number of Validation Images	Number of Test Images	Total
Apple Black Rot	1661	158	79	1900
Apple Healthy	2215	211	105	2531
Apple Rust	1500	151	76	1727
Apple Scab	1269	113	56	1438
Total	6645	633	316	7594

**Table 2:** Performance comparison of different models.

Model	Precision	Recall	mAP@50	mAP@50-95
YOLOv8	0.752	0.851	0.838	0.765
YOLOv9t	0.132	0.328	0.123	0.066
YOLOv10n	0.309	0.486	0.399	0.361
YOLOv11	0.591	0.543	0.447	0.412
YOLOv11_DSSA	**0.973**	**0.978**	**0.991**	**0.949**

## Data Availability

Data are contained within the article.
